# Characteristics and growth of the genetic HIV transmission network of Mexico City during 2020

**DOI:** 10.1002/jia2.25836

**Published:** 2021-11-11

**Authors:** Vanessa Dávila‐Conn, Claudia García‐Morales, Margarita Matías‐Florentino, Eduardo López‐Ortiz, Héctor E. Paz‐Juárez, Ángeles Beristain‐Barreda, Miroslava Cárdenas‐Sandoval, Daniela Tapia‐Trejo, Dulce M. López‐Sánchez, Manuel Becerril‐Rodríguez, Pedro García‐Esparza, Israel Macías‐González, Patricia Iracheta‐Hernández, Steven Weaver, Joel O. Wertheim, Gustavo Reyes‐Terán, Andrea González‐Rodríguez, Santiago Ávila‐Ríos

**Affiliations:** ^1^ Centre for Research in Infectious Diseases National Institute of Respiratory Diseases Mexico City Mexico; ^2^ Clínica Especializada Condesa Mexico City Mexico; ^3^ Institute for Genomics and Evolutionary Medicine Temple University Philadelphia Pennsylvania USA; ^4^ Department of Medicine University of California San Diego La Jolla California USA; ^5^ Coordinating Commission of the National Institutes of Health and High Specialty Hospitals Mexico City Mexico

**Keywords:** molecular epidemiology, genetic clustering, transmission network, HIV prevention, public health, Mexico

## Abstract

**Introduction:**

Molecular surveillance systems could provide public health benefits to focus strategies to improve the HIV care continuum. Here, we infer the HIV genetic network of Mexico City in 2020, and identify actively growing clusters that could represent relevant targets for intervention.

**Methods:**

All new diagnoses, referrals from other institutions, as well as persons returning to care, enrolling at the largest HIV clinic in Mexico City were invited to participate in the study. The network was inferred from HIV *pol* sequences, using pairwise genetic distance methods, with a locally hosted, secure version of the HIV‐TRACE tool: Seguro HIV‐TRACE. Socio‐demographic, clinical and behavioural metadata were overlaid across the network to design focused prevention interventions.

**Results:**

A total of 3168 HIV sequences from unique individuals were included. One thousand and one‐hundred and fifty (36%) sequences formed 1361 links within 386 transmission clusters in the network. Cluster size varied from 2 to 14 (63% were dyads). After adjustment for covariates, lower age (adjusted odds ratio [aOR]: 0.37, *p*<0.001; >34 vs. <24 years), being a man who has sex with men (MSM) (aOR: 2.47, *p* = 0.004; MSM vs. cisgender women), having higher viral load (aOR: 1.28, *p*<0.001) and higher CD4+ T cell count (aOR: 1.80, *p*<0.001; ≥500 vs. <200 cells/mm^3^) remained associated with higher odds of clustering. Compared to MSM, cisgender women and heterosexual men had significantly lower education (none or any elementary: 59.1% and 54.2% vs. 16.6%, *p*<0.001) and socio‐economic status (low income: 36.4% and 29.0% vs. 18.6%, *p* = 0.03) than MSM. We identified 10 (2.6%) clusters with constant growth, for prioritized intervention, that included intersecting sexual risk groups, highly connected nodes and bridge nodes between possible sub‐clusters with high growth potential.

**Conclusions:**

HIV transmission in Mexico City is strongly driven by young MSM with higher education level and recent infection. Nevertheless, leveraging network inference, we identified actively growing clusters that could be prioritized for focused intervention with demographic and risk characteristics that do not necessarily reflect the ones observed in the overall clustering population. Further studies evaluating different models to predict growing clusters are warranted. Focused interventions will have to consider structural and risk disparities between the MSM and the heterosexual populations.

## 1 INTRODUCTION

The fast intra‐host evolution of HIV leads to the accumulation of genetic diversity [1, 2, 3, 4]. This high diversity opens the opportunity of inferring local HIV transmission networks. Annotating the observed genetic clusters with demographic, clinical and behavioural metadata could enrich the design of focused prevention interventions, guiding the allocation of scarce resources to produce higher public health impact [5, 6, 7]. Several approaches for the prioritization of HIV genetic clusters for intervention have been reported, ranging from identification of rapidly growing clusters [8], clusters with incident cases [9, 10], individuals with specific transmission risk characteristics, such as high network connectivity, use of injectable drugs, belonging to specific age groups or with specific sexual practices [11, 12, 13, 14, 15], to the development of combined transmissibility scores [6] or modelling [14, 16, 17, 18].

The HIV epidemic in Mexico is concentrated in key populations [19, 20, 21, 22, 23, 24, 25]. Although the role of heterosexual transmission increasingly plays an important role at the country level, the epidemic in the metropolitan zone of Mexico City remains highly concentrated in men who have sex with men (MSM) [22]. The Condesa Clinic, with two operational branches within Mexico City, is one of the largest HIV care facilities in Latin America, with over 18,000 clients. The clinic is also a major centre for HIV testing in central Mexico, diagnosing nearly 3500 new infections in 2020 (92% cisgender men, 6% cisgender women and 2% transgender men), that represent over a fourth of all new HIV diagnoses in the country and 70–80% of diagnoses in the metropolitan area of Mexico City [26]. Since 2016, the Center for Research in Infectious Diseases of the National Institute of Respiratory Diseases (CIENI/INER), a reference laboratory for HIV genotyping, works with the Condesa Clinic to perform HIV drug‐resistance surveillance [27]. Baseline HIV genotyping is not standard‐of‐care in Mexico [28] and this collaboration represents an additional effort to improve the HIV care continuum locally.

Here, we infer the HIV genetic network of Mexico City in 2020, and identify and describe actively growing clusters that could represent relevant targets for intervention.

## 2 METHODS

### 2.1 Study population

All persons newly admitted at Condesa Clinic, including new diagnoses, referrals from other institutions and persons returning to care, were invited to participate. These inclusion criteria were defined according to the World Health Organization recommendations for performing pre‐treatment drug‐resistance surveillance [29]. Participants answered a computer‐based, self‐administered questionnaire, including socio‐demographic, clinical and behavioural metadata. Paper‐based questionnaires were available for persons preferring not to use the computer‐based option. Participants then donated a blood sample for HIV genotyping. Enrolment took place from July 2019 to December 2020, excluding April to June 2020, when HIV services were temporarily restricted due to the coronavirus disease 19 (COVID‐19) epidemic. The study was approved by the INER institutional review board (project code E02‐20). Participants provided written informed consent to use sequencing data both for HIV drug resistance and HIV genetic network studies.

### 2.2 HIV sequencing

A fragment, including the complete HIV *gag* and *pol* genes (5462 bp; HXB2 positions: 769–6231), was amplified and sequenced using standard next‐generation sequencing (NGS) techniques (Illumina, San Diego, CA) (see Supplementary Methods). Reads were filtered and assembled using HyDRA Web (Public Health Agency of Canada) [30, 31]. Twenty percent consensus sequences were generated and used in HIV drug resistance and clustering analyses. This threshold has been previously defined to provide an excellent agreement between NGS and standard Sanger sequencing [32]. The sequence database was curated, excluding duplicates, as well as sequences not compliant with quality control [33]. Briefly, sequences were excluded due to inadequate length, presence of stop codons, bad insertions/deletions, excess Apolipoprotein B mRNA‐Editing Catalytic Polypeptide‐like (APOBEC) or unusual mutations.

### 2.3 Clustering analyses

Clusters were defined by a genetic distance matrix method, using Seguro HIV‐TRAnsmission Cluster Engine (Seguro HIV‐TRACE), a locally adapted and secured version of the HIV‐TRACE tool [34]. Seguro HIV‐TRACE, like the Secure HIV‐TRACE implemented for U.S. public health departments, permits the analysis and storage of HIV transmission clusters accessible only to registered users. The HIV‐TRACE display was translated into Spanish and adapted to include the variables of interest and fulfil the local data security requirements (see Supporting Information). Clusters were defined as sequences with pairwise Tamura‐Nei 93 genetic distance <0.015. This threshold has been previously demonstrated to be in line with the expected divergence between sequences within an individual [35] and in accordance with the genetic distance between named HIV‐risk partners [36, 37]. Georeferencing of participants according to municipality and zip code of residence was performed according to the National Institute of Statistics and Geography (INEGI) coding, using the program QGIS v3.16.

To identify clusters with active growth, we divided the study period into five 3‐month stages and looked for new clustering cases during each stage: (1) July–September 2019, (2) October–December 2019, (3) January–March 2020, (4) July–September 2020 and (5) October–December 2020; from April to June 2020, no enrolment took place due to limitations imposed by the COVID‐19 sanitary emergency. Clusters with constant growth were defined as those with at least one node added across each stage; new clusters, as those formed during the last two stages; clusters with growth reactivation, as those with at least one node added during the last stage and no growth in previous stages; and clusters with no recent growth as those with no nodes added during the last stage.

### 2.4 Statistical analyses

Data were systematically collected and stored in a local server. Socio‐demographic variables included age, nationality, place of residence, sex at birth, gender identity, civil status, religion, ethnicity, education level, employment, monthly income, self‐perceived social class and use of public transport. Clinical variables included HIV‐infected partners, previous HIV tests, antiretroviral treatment (ART) exposure, diagnosed sexually transmitted infection, circumcision, HIV viral load and CD4+ T cell count. Behavioural variables included sexual preference, age of sexual onset, risk sexual practices, drug use, travel, use of apps to find sexual partners, venues and commercial sex. All time‐related variables (including CD4+ T cell count, viral load and place of residence) were current at the time of HIV testing, coinciding with the time of enrolment, HIV sequencing and HIV diagnosis for the majority of the participants (80%). For continuous variables, descriptive statistical parameters were expressed as median and interquartile range. Age was categorized in quartiles in order to identify specific strata amenable to intervention; CD4+ T cell count was stratified according to clinical criteria. Categorical variables were expressed as frequencies or percentages and 95% confidence interval. Exploratory and bivariate data analyses were performed comparing sexual risk groups, as well as persons within clusters versus not in clusters. The sexual risk variable was constructed based on sex at birth, gender identity and sexual preferences. Mann–Whitney U test was used to compare continuous variables and chi^2^ test or Fisher's exact test for categorical variables. Multivariable logistic regression models for clustering risk were constructed including statistically significant variables from the bivariate analysis. The best model was selected verifying the fit of the model using Akaike information criterion, Bayesian information criterion and Hosmer–Lemeshow goodness‐of‐fit test (Table S1). All analyses were performed using STATA v16.

## 3 RESULTS

### 3.1 Network characteristics

A total of 3419 individuals were enrolled, from whom 3195 (93.4%) HIV sequences were successfully obtained. After curation of the database and removal of duplicates, 3168 (99.2%) HIV sequences from different individuals were included; 92.8% were cisgender men, 5.5% were cisgender women, 1.6% were transgender women and 0.1% were transgender men; 80.7% resided in Mexico City, and 17.3% in the neighbouring state of Mexico. For sexual risk stratification, we observed an important proportion of missing information (60%), making it difficult to distinguish heterosexual cisgender men and MSM. Using the data available, MSM were associated with higher education (none or any elementary: 16.6% vs. 54.2% and 59.1%; *p*<0.001) and higher socio‐economic level (monthly income <$4000 pesos: 18.6% vs. 29.0% and 36.4%; *p* = 0.03) than heterosexual cisgender men and women. Cisgender women and heterosexual cisgender men exhibited lower CD4+ T cell counts than MSM (*p*<0.001). Age distribution also varied between sexual risk groups, with MSM being generally younger than cisgender women, heterosexual cisgender men and transgender women (*p*<0.001) (Table 1).

**Table 1:** General characteristics of participants by sexual risk category^a^

	MSM *n* = 1039		Transgender women *n* = 33		Heterosexual cisgender men *n* = 108		Cisgender women *n* = 88		*p* value
	*n*	%	*n*	%	*n*	%	*n*	%	
Age (years)									
<24	213	20.50	6	18.18	17	15.74	14	15.91	<0.001*
24–27	240	23.10	9	27.27	20	18.52	11	12.50	
28–34	347	33.40	6	18.18	19	17.59	21	23.86	
>34	239	23.00	12	36.36	52	48.15	42	47.73	
Social security									
Yes	255	24.69	2	6.25	19	17.59	7	8.14	0.003*
No	704	68.15	27	84.38	79	73.15	70	81.40	
Unknown	74	7.16	3	9.38	10	9.26	9	10.47	
State of residence									
Mexico City	777	74.78	30	90.91	85	78.70	69	78.41	0.099
Mexico State	240	23.10	2	6.06	18	16.67	16	18.18	
Other	22	2.12	1	3.03	5	4.63	3	3.41	
Nationality									
Mexican	1003	96.91	30	90.91	105	99.06	88	100.00	0.032*
Foreign	32	3.09	3	9.09	1	0.94	0	0.00	
Time in current residence									
<1 year	214	20.66	9	27.27	12	11.32	11	12.50	<0.001*
1–10 years	328	31.66	14	42.42	22	20.75	16	18.18	
>10 years	494	47.68	10	30.30	72	67.92	61	69.32	
Travel in the last 6 months									
Yes	334	33.50	9	28.13	30	29.41	20	24.39	0.304
No	663	66.50	23	71.88	72	70.59	62	75.61	
Persons cohabiting									
Partner	117	11.29	1	3.13	15	14.02	9	10.59	<0.001*
Family	636	61.39	13	40.63	76	71.03	69	81.18	
Alone	113	10.91	8	25.00	9	8.41	4	4.71	
Other	170	16.41	10	31.25	7	6.54	3	3.53	
Civil status									
Partnered/married	160	15.63	2	6.25	39	39.39	31	39.24	<0.001*
Single	864	84.38	30	93.75	60	60.61	48	60.76	
Indigenous language									
Yes	29	2.79	1	3.03	8	7.41	4	4.65	0.072
No	1009	97.21	32	96.97	100	92.59	82	95.35	
Monthly income (pesos)^b^									
<$4000	92	18.59	3	42.86	9	29.03	8	36.36	0.032*
$4000–$11,000	290	58.59	3	42.86	20	64.52	13	59.09	
>$11,000	113	22.83	1	14.29	2	6.45	1	4.55	
Self‐perceived social class									
Low	361	34.78	19	57.58	62	57.41	51	59.30	<0.001*
Medium	675	65.03	14	42.42	46	42.59	35	40.70	
High	2	0.19	0	0.00	0	0.00	0	0.00	
Partner's HIV diagnosis									
Without HIV	110	10.72	1	3.03	21	20.39	15	17.05	0.004*
With HIV	176	17.15	3	9.09	11	10.68	15	17.05	
Unknown	176	17.15	7	21.21	19	18.45	29	32.95	
No partner	564	54.97	22	66.67	52	50.49	29	32.95	
Education									
None or any elementary	172	16.60	22	66.67	58	54.21	52	59.09	< 0.001*
High school	403	38.90	9	27.27	35	32.71	22	25.00	
Career	461	44.50	2	6.06	14	13.08	14	15.91	
Occupation									
Employed	498	48.35	11	34.38	48	45.71	31	35.63	<0.001*
Unemployed	257	24.95	12	37.50	31	29.52	17	19.54	
Student	95	9.22	0	0.00	6	5.71	2	2.30	
Domestic labour	17	1.65	3	9.38	2	1.90	19	21.84	
Other	72	6.99	4	12.50	10	9.52	10	11.49	
>1 Job	91	8.83	2	6.25	8	7.62	8	9.20	
Use of public transport									
Bus	816	78.76	14	43.75	70	66.67	59	70.24	<0.001*
Trolleybus	447	43.15	0	0.00	24	22.86	6	7.14	
Taxi	376	36.29	8	25.00	34	32.38	9	10.71	
Metrobus (BRT)	73	7.05	3	9.38	1	0.95	12	14.29	
Subway	191	18.44	20	62.50	12	11.43	36	42.86	
Other	37	3.57	6	18.75	4	3.81	7	8.33	
First HIV test									
Yes	332	31.98	11	33.33	44	40.74	28	32.56	0.688
No	685	65.99	21	63.64	62	57.41	57	66.28	
Prefer not to answer	21	2.02	1	3.03	2	1.85	1	1.16	
Date of last HIV test									
≤6 months	262	39.28	5	26.32	26	41.94	29	51.79	0.175
>6 months	405	60.72	14	73.68	36	58.06	27	48.21	
Last HIV test result									
Positive	327	47.67	15	71.43	43	69.35	43	76.79	<0.001*
Negative	334	48.69	6	28.57	11	17.74	9	16.07	
Indeterminate	11	1.60	0	0.00	5	8.06	0	0.00	
Unknown	14	2.04	0	0.00	3	4.84	4	7.14	
Recent infection (≤6 months)^c^									
Yes	64	6.29	1	3.13	2	1.89	3	3.53	0.194
No	953	93.71	31	96.88	104	98.11	82	96.47	
Other STI in the last 6 months									
Yes	395	39.98	7	24.14	29	28.43	35	43.75	0.034*
No	593	60.02	22	75.86	73	71.57	45	56.25	
Use of injectable drugs									
Yes	50	4.84	0	0.00	4	3.74	1	1.16	0.233
No	983	95.16	33	100.00	103	96.26	85	98.84	
CD4+ T cell count (cells/mm^3^)									
<200	528	50.87	11	33.33	78	72.90	56	63.64	<0.001*
200–499	448	43.16	18	54.55	26	24.30	25	28.41	
≥500	62	5.97	4	12.12	3	2.80	7	7.95	

Abbreviations: BRT, bus rapid transit; STI, sexually transmitted infections.

^a^ Sexual risk was assessed as a composite variable, including sex at birth, gender identity and sexual preference, and was available for 40% of the total 3168 participants. All time‐associated variables, including CD4+ T cell count and place of residence, refer to the time of HIV testing, HIV sequencing and enrolment in the study. Column percentages are shown without considering missing data.

^b^ Monthly family income was stratified according to the National Council of Evaluation of Social Development Policy (CONEVAL); <$4000 indicates poverty and >$11,000 is considered high in urban zones.

^c^ Reporting a negative HIV test 6 months prior to diagnosis.

* Statistically significant.

**Figure 1:**
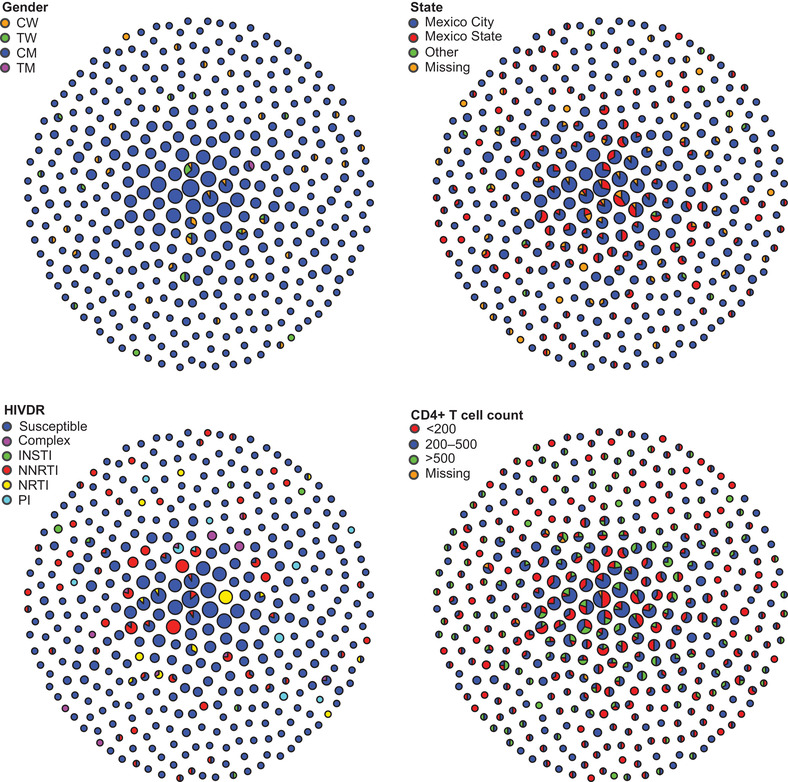
HIV genetic network in Mexico City's metropolitan zone, 2020. The network was inferred from 3168 viral pol sequences, from which 1150 (36%) formed links within 386 clusters from 2 to 14 nodes. Clusters are shown compacted. Each circle represents a cluster. Circle size represents cluster size. Each panel shows the network coloured by different variables of interest. The network was inferred using Seguro HIV‐TRACE as described in the Methods. CD4+ T cell counts are expressed in cells/mm^3^. Abbreviations: CM, cisgender men; CW, cisgender women; INSTI, integrase strand transfer inhibitors; NNRTI; non‐nucleoside reverse transcriptase inhibitors; NRTI, nucleoside reverse transcriptase inhibitors; PI, protease inhibitors; TM, transgender men; TW, transgender women.

Out of 3168 sequences, 1150 (36.3%) formed 1361 links within 386 transmission clusters in the network. Cluster size ranged from 2 to 14 (242/386 were dyads). Considering only clustering persons, 95.3% were cisgender men, 2.9% were cisgender women, 1.7% were transgender women and 0.2% were transgender men; 80.9% of clustering persons resided in Mexico City; 33.5% had <200, 51.8% 200–499 and 14.7% ≥500 CD4+ T cells/mm^3^ at enrolment; 11.1% showed resistance to non‐nucleoside reverse transcriptase inhibitors, 4.1% to nucleoside reverse transcriptase inhibitors, 3.5% to protease inhibitors and 0.9% to integrase inhibitors (Figure 1). The number of links per node ranged from 1 to 12 with 242/1361 (18%) of edges belonging to dyads. Considering clusters with three or more individuals, the most common type of genetic link was between two cisgender men (1064/1119, 95.1%) and the least frequent was between two cisgender women (1/1119, 0.1%). Transgender persons were members of 18/1119 (1.6%) links.

**Figure 2:**
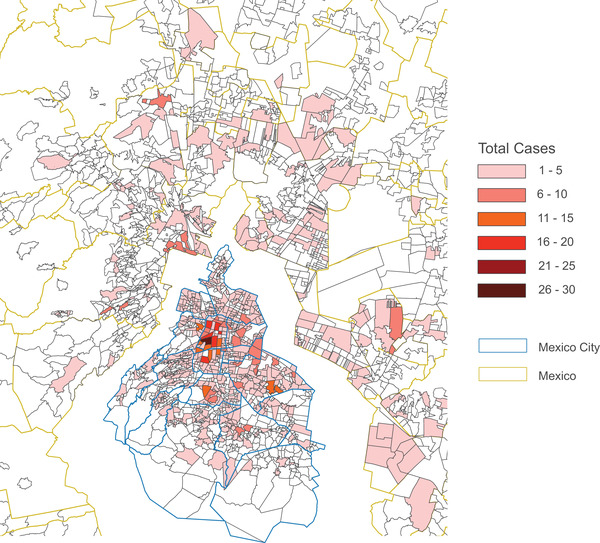
Geographical distribution of participants in Mexico City's metropolitan zone. The map shows distribution of participants by zip code of residence at the time of enrolment, as a heat map. Mexico City municipalities (blue) and the neighbouring municipalities of the state of Mexico (yellow) are shown.

Considering participants with data on municipality of residence available (2042), five municipalities in Mexico City comprised 43%: Cuauhtémoc (14.2%), Iztapalapa (10.0%), Gustavo A. Madero (8.4%), Benito Juárez (5.4%) and Coyoacán (5.3%). The same municipalities included 44% of persons belonging to clusters (724 with available data). The 10 most frequent zip codes of residence were concentrated in two municipalities of Mexico City (839 cases with data available): eight in Cuauhtémoc (18%) and two in Benito Juárez (4%). Considering only clustering individuals (636 cases with data available), the 10 most frequent zip codes were concentrated in four municipalities: five in Cuauhtémoc (5%), two in Benito Juárez (3%), two in Coyoacán (2%) and one in Iztacalco (1%) (Figure 2).

### 3.2 Characteristics of individuals within clusters

Comparing clustering versus non‐clustering individuals, we observed significant differences in median age (27 vs. 29 years; *p*<0.001), proportion of cisgender men (95.3% vs. 91.4%; *p*<0.001; including both heterosexual and MSM), proportion of MSM (91.2% vs. 81.3%; *p*<0.001), education level (none or any elementary: 20.8% vs. 26.6%; *p* = 0.04), previous negative HIV test result, if performed (51.7% vs. 38.6%; *p* = 0.005), recent infection (previous negative HIV test within 6 months) (7.5% vs. 4.6%; *p* = 0.029), viral load median (50,565 vs. 45,642 copies/ml; *p* = 0.032), CD4+ T cell count median (273 vs. 211 cells/mm^3^; *p*<0.001) and the use of venues for sex (31.2% vs. 25.2%; *p* = 0.04) (Table 2).

**Table 2:** Socio‐demographic, clinical and behavioural characteristics of participants within clusters in Mexico City's HIV transmission network 2020

		Total *n*	Clustering *n* (%)^a^	OR	95% CI			*p* value
Age (years)	<**24**	**620**	**299 (26.07)**			**Ref**.		
	24–27	764	308 (26.85)	0.73	0.59	–	0.90	<0.001*
	29–34	985	354 (30.86)	0.60	0.49	–	0.74	<0.001*
	>34	795	186 (16.22)	0.33	0.26	–	0.41	<0.001*
Gender identity	**Cisgender women**	**173**	**33 (2.87)**			**Ref**.		
	Cisgender men	2941	1096 (95.3)	2.52	1.71	–	3.71	<0.001*
	Transgender women	51	19 (1.65)	2.52	1.27	–	4.99	0.008*
	Transgender men	3	2 (0.17)	8.48	0.75	–	96.40	0.085
State of residence	**Mexico City**	**2417**	**875 (80.94)**			**Ref**.		
	Mexico State	519	191 (17.67)	1.03	0.84	–	1.25	0.797
	Other	59	15 (1.39)	0.60	0.33	–	1.09	0.092
Municipality Mexico City	**Cuauhtémoc**	**113**	**42 (8.05)**			**Ref**.		
	Iztapalapa	65	27 (5.17)	1.20	0.64	–	2.24	0.565
	Gustavo A. Madero	21	4 (0.77)	0.40	0.13	–	1.26	0.117
	Other	1302	449 (86.01)	0.89	0.60	–	1.33	0.566
Municipality Mexico State	**Ecatepec**	**80**	**27 (14.21)**			**Ref**.		
	Nezahualcóyotl	54	17 (8.95)	0.90	0.43	–	1.89	0.784
	Naucalpan	48	21 (11.05)	1.53	0.73	–	3.18	0.259
	Tlalnepantla	43	17 (8.95)	1.28	0.60	–	2.76	0.524
	Other	291	108 (56.84)	1.16	0.69	–	1.95	0.580
Travel (previous 6 months)	**No**	**838**	**273 (67.74)**			**Ref**.		
	Yes	394	130 (32.26)	1.02	0.79	–	1.31	0.884
Civil status	**Partnered/married**	**238**	**74 (17.45)**			**Ref**.		
	Single	1043	350 (82.55)	1.12	0.83	–	1.52	0.466
Partner's HIV diagnosis	**Without HIV**	**149**	**43 (10.34)**			**Ref**.		
	With HIV	207	80 (19.23)	1.55	0.99	–	2.44	0.056
	Unknown	237	83 (19.95)	1.33	0.85	–	2.07	0.209
	No partner	691	210 (50.48)	1.08	0.73	–	1.59	0.712
Sexual preference	**Women**	**108**	**24 (5.74)**			**Ref**.		
	Men	1076	368 (88.04)	1.82	1.14	–	2.91	0.013*
	Bisexual	88	26 (6.22)	1.47	0.77	–	2.80	0.243
Education	**None or any elementary**	**323**	**88 (20.75)**			**Ref**.		
	High school	482	172 (40.57)	1.48	1.09	–	2.02	0.012*
	Career	504	164 (38.68)	1.29	0.95	–	1.75	0.107
Employment	**Employed**	**606**	**195 (46.43)**			**Ref**.		
	Unemployed	325	98 (23.33)	0.91	0.68	–	1.22	0.526
	Student	106	37 (8.81)	1.13	0.73	–	1.74	0.581
	Domestic labour	43	8 (1.9)	0.48	0.22	–	1.06	0.069
	Other	100	37 (8.81)	1.24	0.80	–	1.92	0.342
	>1 Job	109	45 (10.71)	1.48	0.98	–	2.25	0.065
Monthly income (pesos)^b^	**<$4000**	**115**	**46 (22.33)**			**Ref**.		
	$4000–$11,000	332	124 (60.19)	0.89	0.58	–	1.38	0.614
	>$11,000	118	36 (17.48)	0.66	0.38	–	1.13	0.130
Persons cohabiting	**Alone**	**137**	**36 (8.61)**			**Ref**.		
	Partner	144	46 (11)	1.32	0.79	–	2.21	0.297
	Family	811	281 (67.22)	1.49	0.99	–	2.23	0.056
	Roommates	174	52 (12.44)	1.20	0.73	–	1.97	0.483
	Other	16	3 (0.72)	0.65	0.17	–	2.40	0.516
Place where most time is spent	**Work**	**510**	**183 (43.26)**			**Ref**.		
	Home	707	213 (50.35)	0.77	0.60	–	0.98	0.035*
	School	35	13 (3.07)	1.06	0.52	–	2.15	0.881
	Other	36	14 (3.31)	1.14	0.57	–	2.28	0.717
Use of public transport	Bus	974	327 (78.61)	1.17	0.92	–	1.49	0.196
	Trolleybus	479	167 (40.14)	1.18	0.93	–	1.50	0.172
	Taxi	430	152 (36.54)	1.21	0.95	–	1.55	0.129
	Metrobus (BRT)	89	32 (7.69)	1.18	0.75	–	1.85	0.478
	Subway	263	90 (21.63)	1.10	0.83	–	1.46	0.517
Indigenous language	**No**	**1261**	**409 (96.01)**			**Ref**.		
	Yes	42	17 (3.99)	1.42	0.76	–	2.65	0.277
Self‐perceived social class	**Low**	**518**	**165 (38.73)**			**Ref**.		
	Medium	783	261 (61.27)	1.07	0.84	–	1.36	0.578
First HIV test	**No**	**843**	**272 (63.85)**			**Ref**.		
	Yes	427	140 (32.86)	1.02	0.80	–	1.31	0.851
	Prefer not to answer	33	14 (3.29)	1.55	0.76	–	3.13	0.225
Date of last HIV test	**≤ 6 months**	**325**	**118 (44.19)**			**Ref**.		
	>6 months	492	149 (55.81)	0.76	0.57	–	1.03	0.073
Recent HIV infection (≤6 months)^c^	**No**	**1200**	**381 (92.48)**			**Ref**.		
	Yes	70	31 (7.52)	1.71	1.05	–	2.78	0.031*
Use of injectable drugs	**No**	**1242**	**409 (96.46)**			**Ref**.		
	Yes	55	15 (3.54)	0.76	0.42	–	1.40	0.383
Other STI in the last 6 months	**No**	**744**	**240 (62.34)**			**Ref**.		
	Yes	475	145 (37.66)	0.92	0.72	–	1.18	0.526
CD4+ T cell count (cells/mm^3^)	**<200**	**1344**	**383 (33.48)**			**Ref**.		
	200–499	1402	593 (51.84)	1.84	1.57	–	2.16	<0.001*
	≥500	405	168 (16.69)	1.78	1.41	–	2.24	<0.001*
Age of sexual initiation^d^		**1249**	**N/A**	1.01	0.98	–	1.05	0.394
Sexual role	**Active**	**242**	**73 (19.57)**			**Ref**.		
	Passive	337	115 (30.83)	1.20	0.84	–	1.71	0.316
	Inter	512	185 (49.6)	1.31	0.94	–	1.82	0.107
Circumcision	**No**	**863**	**299 (78.68)**			**Ref**.		
	Yes	255	81 (21.32)	0.88	0.65	–	1.18	0.394
Use of apps to find sex partners	**No**	**518**	**166 (43.92)**			**Ref**.		
	Yes	584	212 (56.08)	1.21	0.94	–	1.55	0.138
Venues for sex	**No**	**775**	**247 (68.8)**			**Ref**.		
	Yes	290	112 (31.2)	1.35	1.02	–	1.78	0.038*
Sexual risk^e^	**Cisgender women**	**88**	**13 (14.77)**			**Ref**.		
	MSM	1039	366 (35.23)	3.14	1.72	–	5.73	<0.001*
	Transgender women	33	15 (45.45)	4.81	1.95	–	11.87	0.001*
	Heterosexual cisgender men	108	24 (22.22)	1.65	0.78	–	3.47	0.188
	Missing	1900	732 (38.53)	3.62	1.99	–	6.56	<0.001*
Viral load (log copies/ml)^d^		3162	N/A	1.14	1.04	–	1.24	0.004*
Any HIV drug resistance	**No**	**2572**	**948 (82.43)**			**Ref**.		
	Yes	596	202 (17.57)	0.88	0.73	–	1.06	0.175
HIV drug resistance by drug class	**Susceptible**	**2572**	**948 (82.43)**			**Ref**.		
	NRTI	67	28 (2.43)	1.23	0.75	–	2.01	0.410
	NNRTI	371	120 (10.43)	0.82	0.65	–	1.03	0.091
	INSTI	27	9 (0.78)	0.86	0.38	–	1.91	0.706
	PI	61	25 (2.17)	1.19	0.71	–	1.99	0.510
	More than one class	70	20 (1.74)	0.69	0.41	–	1.16	0.158

Abbreviations: BRT, bus rapid transit; CI, confidence interval; INSTI, integrase strand transfer inhibitors; MSM, men who have sex with men; NNRTI; non‐nucleoside reverse transcriptase inhibitors; NRTI, nucleoside reverse transcriptase inhibitors; OR, crude odds ratio; PI, protease inhibitors; Ref., reference category; STI, sexually transmitted infection.

^a^ Column percentages are shown (without considering missing data unless stated).

^b^ Monthly family income was stratified according to the National Council of Evaluation of Social Development Policy (CONEVAL); <$4000 indicates poverty and >$11,000 is considered high in urban zones.

^c^ Reporting a negative HIV test 6 months prior to diagnosis.

^d^ Analysed as continuous variables.

^e^ Constructed as a composite variable considering sex at birth, gender identity and sexual preference.

* Statistically significant.

Assessing crude associations with belonging to clusters, cisgender men had higher odds of clustering than cisgender women (odds ratio [OR]: 2.52, *p*<0.001) and MSM had higher odds of clustering than heterosexual men (OR: 1.90, *p* = 0.006). Persons with recent infection, higher log viral load, higher CD4+ T cell count, higher education and using venues for sex had higher odds of clustering. Persons spending more time at home (compared to work), and of older age, had lower odds of clustering (Table 2). After adjustment for confounders, lower age (adjusted odds ratio [aOR]: 0.37, *p*<0.001; >34 vs. <24 years), being an MSM (aOR: 2.47, *p* = 0.004; vs. cisgender women), being a transgender woman (aOR: 3.81, *p* = 0.005; vs. cisgender women), having higher viral load (aOR: 1.28, *p*<0.001) and having higher CD4+ T cell count (aOR: 1.80, *p*<0.001; ≥500 vs. <200 cells/mm^3^) remained associated with higher adjusted odds of clustering (Table 3). No associations were found stratifying the model by sexual risk categories. Belonging to larger clusters (>5 nodes) among clustering individuals was associated only with age (aOR: 0.55, 0.33–0.90, *p* = 0.02; >34 vs. <24) after adjusting for sexual risk, viral load and CD4+ T cell count.

**Table 3:** Multivariable analysis of risk of belonging to clusters in Mexico City's HIV transmission network, 2020

		*n*	% Clustering^a^	aOR	*p* value	95% CI		
Age (years)	**<24**	**620**	**26.07**		**Ref**.			
	24–27	764	26.85	0.72	0.003	0.58	–	0.89
	28–34	985	30.86	0.61	<0.001*	0.50	–	0.75
	>34	795	16.22	0.37	<0.001*	0.29	–	0.46
Sexual risk category^b^	**Cisgender women**	**88**	**1.13**		**Ref**.			
	MSM	1039	31.83	2.47	0.004*	1.34	–	4.56
	Transgender women	33	1.30	3.81	0.005*	1.51	–	9.61
	Heterosexual cisgender men	108	2.09	1.63	0.206	0.76	–	3.47
	Missing	1900	63.65	2.58	0.002*	1.40		4.73
Viral load (log copies/ml)^c^		3162	N/A	1.28	<0.001*	1.16	–	1.40
CD4+ T cell count (cells/mm^3^)	**<200**	**1344**	**33.48**		**Ref**.			
	200–499	1402	51.84	1.73	<0.001*	1.46	–	2.05
	≥500	405	14.69	1.80	<0.001*	1.40	–	2.31

Abbreviations: aOR, adjusted odds ratio; CI, confidence interval; MSM, men who have sex with men; Ref., reference category.

^a^ Row percentages are shown (without considering missing data, unless stated).

^b^ Sexual risk category was assessed as a composite variable, including sex at birth, gender identity and sexual practices. Due to the considerable proportion of missing data, these are shown as a separate category.

^c^ Analysed as a continuous variable.

* Statistically significant.

### 3.3 Identification of actively growing clusters

**Figure 3:**
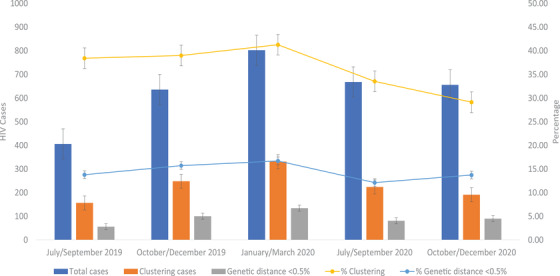
Enrolment and clustering rate across the study period. The total number of participants, number of participants forming clusters and number of individuals participating in links with genetic distance <0.5%, suggesting direct and recent transmission are shown by trimester of the study period. Clustering rate and proportion of links with genetic distance <0.5% are also shown. Clustering persons were defined considering the final network in the complete study period. Dispersion measures indicate 95% confidence intervals.

Four hundred and six participants were enrolled during the first 3‐month stages (as defined in the Methods), with 156 persons forming clusters (38.4%). The clustering rates during the following 3‐month periods were 248/636 (39.0%), 331/802 (41.3%), 224/668 (33.5%) and 191/656 (29.1%), respectively (Figure 3). We observed a decreasing trend in clustering rate towards the end of 2020 after the onset of the COVID‐19 pandemic (fifth vs. third period: *p*<0.0001) (Figure 3). The proportion of participants with at least one link with genetic distance <0.5% did not vary significantly across time stages.

We observed a significant mean age increase comparing clustering individuals in the second (29.8 years, standard deviation [SD] 8.9) versus the fifth stage (31.6 years, SD 9.3) (*p* = 0.002). Considering clusters with three or more nodes, the most frequent links included persons of the third age quartile (28–34 years) (31.4%), followed by the first (<24 years) (30.2%). We observed 201/1361 (14.8%) links between persons with 10 or more years of difference in age. The proportion of links with genetic distance <0.5% varied across age quartiles: 23%, 22%, 17% and 29% in persons <24, 24–27, 28–34 and >34 years, respectively, and was significantly higher in persons >34 years compared to all other age strata (*p*<0.05). The average number of links per node (grade) showed a decreasing trend by age quartiles for MSM (2.9, 2.3, 2.4 and 1.7), but not for cisgender women (1.5, 3.0, 2.2 and 1.0) or heterosexual cisgender men (1.3, 2.0, 1.5 and 1.3).

**Figure 4:**
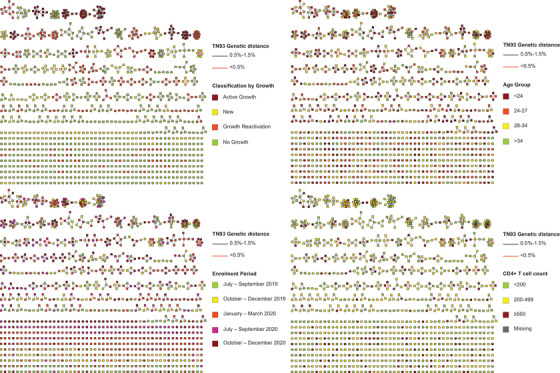
Identification of priority transmission clusters in the HIV genetic network of Mexico City, 2020. Clusters are shown ordered by size in each panel. The colour of the nodes corresponds to attributes described in the legends of each panel, including classification by growth, age, enrolment period and CD4+ T cell counts. Links are coloured by genetic distance. The network was inferred using Seguro HIV‐TRACE as described in the Methods. Clusters with active growth are defined as those in which at least one node was added during each time stage (trimester); clusters with recent growth include new clusters appearing during the last two enrolment stages, clusters with growth reactivation include clusters in which at least one node was added during the last stage and no growth was observed in previous stages; and clusters without growth as those for which no nodes were added during the last stage. Age is expressed in years and CD4+ T cell counts are expressed in cells/mm^3^. Abbreviation: TN93, Tamura‐Nei genetic distance.

**Figure 5:**
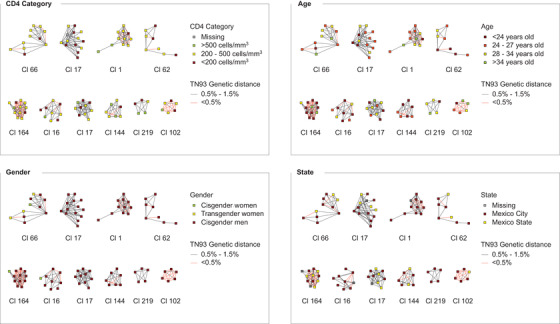
Clusters with active growth. Details of the 10 clusters in which at least one node was added during each 3‐month stage of the study period (as explained in the Methods and shown in Figure 4) are shown. Each panel shows the same 10 clusters coloured by different attributes. The network was inferred using Seguro HIV‐TRACE as described in the Methods. Abbreviation: TN93, Tamura‐Nei genetic distance.

We identified 10 clusters with constant growth (2.6% of all clusters), 61 (15.8%) new clusters, 85 (22.0%) clusters with growth reactivation and 230 (59.6%) clusters with no recent growth (see Methods; Figure 4). Clusters with active growth incorporated an average of 2.1 nodes per time stage. The percentages of cisgender women in clusters with no recent growth, recent formation, growth reactivation and active growth were 3.1%, 2.8%, 2.1% and 3.2%, respectively; for transgender women, 1.2%, 3.4%, 0.9% and 3.2%, respectively. The percentages of persons with CD4+ T cell counts under 200 cells/mm^3^ were 31.0%, 46.3%, 31.5% and 30.1%; and the median age was 28, 29, 28 and 26, respectively. Among the 10 clusters with constant growth, seven involved only cisgender men, two included cisgender women and men, and one included transgender women, cisgender women and men, which suggests sexual risk intersectionality (Figure 5). Clusters 16 and 164 included a majority of cisgender men <24 years. Clusters 164, 17 and 1 included high‐grade nodes and cluster 164 showed the highest number of links with genetic distance <0.5% (11 links). Cluster 164 included highly connected nodes formed by older MSM with low genetic distance links to much younger (at least 10 years) MSM, while cluster 17 included highly connected nodes formed by MSM <24 years. Individuals forming cluster 219 were concentrated in two municipalities of Mexico City, while individuals forming the rest of actively growing clusters showed higher geographical dispersion, with seven of the clusters including persons residing in Mexico State. Also relevant is the presence of clusters with apparent bridge nodes linking possible sub‐clusters (66, 62 and 1) (Figure 5 and Figure S1).

## 4 DISCUSSION

We implemented an HIV molecular surveillance system, Seguro HIV‐TRACE, to construct and monitor the HIV genetic network in Mexico City. The population was enrolled at the largest primary HIV care facility in Mexico. Sampling density was high, with more than a third of the participants belonging to clusters. Clustering rate was similar or even higher to that observed in other large metropolitan areas [12, 14]. However, sampling density in highly populated, geographically delimited settings such as Mexico City could be improved with network‐informed strategies for in‐depth sampling of high‐risk populations, such as MSM [38]. In our study, enrolment bias could exist, as additional diagnoses, mainly in persons with formal employment residing in municipalities where social security clinics are available, could be missed. Also, not all persons diagnosed at the clinic were enrolled due to logistic complications and staff availability, mainly in the pm shift. However, comparing our study database with the database of the clinic, we estimate a significant improvement of the enrolment percentage, from approximately 40% at the beginning of the study period (late 2019), to nearly 80% in the end (late 2020), which could explain differences in sample size in the same months of different years along the study period (Figure 3), and could affect completeness of the network. In spite of this, the socio‐demographic characteristics of the study participants matched expectations, according to previous reports [19, 20, 22, 27].

The overall clustering rate was significantly reduced after the onset of the COVID‐19 pandemic. This observation could reflect behavioural change, mostly within MSM, associated with social distancing or, alternatively, a change in diagnosis trends, with a reduced proportion of young MSM attending the clinic for an HIV test, given the discrete but significant increase in average age of clustering individuals observed towards the end of the study period. Nevertheless, HIV detection and clinical services at the Condesa clinics remained open during the COVID‐19 sanitary emergency, becoming a huge support for other clinics and hospitals that suspended these services. This could have caused possible subtle changes in the demographics of the serviced population that might explain our observations [26].

After adjusting for confounders, we observed that local HIV transmission and network growth was strongly driven by young MSM with recent infection and higher education, which supports previous observations by our group [27], is consistent with the highly concentrated epidemic in the region, and suggests a clear population for immediate intervention. In agreement with previous reports [22], the socio‐demographic characteristics of cisgender women and heterosexual cisgender men in the network contrast with those observed in MSM, being generally older, with lower education and lower socio‐economic status than the latter.

Leveraging network inference to suggest more focused interventions, we identified 10 clusters of immediate interest, given their constant growth across the study period with an average incorporation of two nodes per trimester. Prevention interventions, including pre‐exposure prophylaxis, not yet widely available in Mexico, could be most cost‐effective when focused on persons associated with specific transmission chains represented by clusters such as the ones selected here. Moreover, some of these actively growing clusters represent opportunities for intervention that would be missed if based only on the majoritarian clustering population characteristics. For example, cluster 66 illustrates a transmission chain combining sexual risk categories, including cisgender men (both MSM and heterosexual men), cisgender women and transgender women. This opens the opportunity of targeting contacts of connecting nodes between sexual risk groups, as well as possible hard‐to‐reach individuals not present in the cluster, but suggested by less frequently expected links such as between two transgender women. Also, clusters with high‐grade nodes with different characteristics such as 164, including highly connected older MSM with low genetic distance links to much younger (at least 10 years) MSM, and cluster 17 with highly connected MSM <24 years could represent opportunities to break important transmission chains with the involvement of nodes of unusual characteristics. Also relevant is the presence of clusters with apparent bridge nodes linking sub‐clusters with different assortativity characteristics (66, 62 and 1). This type of cluster topology has been previously identified as possible target for public health intervention [39].

The present study has important limitations. As explained, enrolment bias could exist by including only one clinic and by missed opportunities of enrolment, even if this clinic concentrates the majority of local diagnoses. Although the demographics of the studied population suggests high representativeness with respect to the overall population of persons living with HIV in Mexico City, completeness of the network could be improved by increasing the proportion of newly diagnosed persons participating in the study. Importantly, the completeness of the metadata could be improved, as many variables had a high proportion of missing data. Missing data makes it difficult to further discuss relationships and intersectionality of variables in the context of the network, limiting our conclusions to general overall observations for the clustering population. This is especially true for the sexual risk composite variable, for which nearly 60% of the data were missing, mainly limiting distinction between heterosexual cisgender men and MSM. Additionally, the captured metadata did not allow us to identify persons returning to care, and information on previous exposure to antiretroviral treatment was incomplete and did not allow us to study ART defaulters as a separate group, nor their role within the network. Information bias could also exist, as many participants refused to answer sensitive or private questions given that many had just received their diagnosis. We were able to improve response rates towards the end of the study period by increasing the number of staff overseeing enrolment and by adjusting the moment along the process of linkage to care in which potential participants are invited to participate in the study.

## 5 CONCLUSIONS

The present study suggests that HIV transmission in the metropolitan zone of Mexico City is strongly driven by links between young MSM with higher education level, higher viral load and higher CD4+ T cell counts, suggesting an immediate target group for intervention. Nevertheless, leveraging network inference, we identified actively growing clusters which could be prioritized for focused intervention, that included intersecting sexual risk groups, highly connected nodes with demographic and risk characteristics that do not necessarily reflect the ones observed in the overall clustering population and bridge nodes between possible sub‐clusters with high growth potential. Further studies testing different models to predict growing clusters and evaluating the effectiveness of focused intervention based on prediction of priority clusters versus interventions based on overall characteristics of the clustering population are warranted. Furthermore, interventions will have to consider structural and risk disparities between the MSM and heterosexual populations.

## COMPETING INTERESTS

The authors declare no competing interests.

## AUTHORS’ CONTRIBUTIONS

SAR, GRT and AGR conceived, planned and designed the study. CGM, VDC and ELO analysed the data. MMF performed HIV sequencing. HEPJ implemented the electronic questionnaire for data collection. CGM, DTT, DMLS and PIH coordinated sample collection and processing. VDC, AB, MS, MBR, PGE and IMG coordinated participant enrolment and data collection. SW and JOW designed and implemented Seguro HIV‐TRACE for clustering analyses. SAR wrote the manuscript. All authors have read and approved the final manuscript.

## FUNDING

This work was supported by grants from Consejo Nacional de Ciencia y Tecnología (CONACyT – PRONAII Virología 303079). The work was supported in part by funds from the San Diego Center for AIDS Research International Pilot Grant (P30 AI036214; Sub‐award No. 112605914), the Canadian Institutes of Health Research (grants PJT‐148621 and PJT‐159625) and the Mexican Government (Programa Presupuestal P016; Anexo 13 del Decreto del Presupuesto de Egresos de la Federación). AB was supported by a scholarship from AIDS Healthcare Foundation (AHF) Mexico.

## Supporting information




**Table S1**. Comparison and selection of multivariable models.
**Figure S1**. Summary of characteristics of clusters with active growth.

## Data Availability

All data are available from the corresponding author upon reasonable request.
