# Supplementary material for: Characteristics and growth of the genetic HIV transmission network of Mexico City during 2020
Source: J Int AIDS Soc. 2021 Nov 11;24(11):e25836. doi: 10.1002/jia2.25836 (PMC8583431; doi:10.1002/jia2.25836)
Supplement: Supplementary file 1 — Table S1. Comparison and selection of multivariable models. Figure S1. Summary of characteristics of clusters with active growth. [file JIA2-24-e25836-s001.docx]

**Supplementary Table 1.** Comparison and selection of multivariable models. ^a^

|  |  | Model | | | | | | | | | |
| --- | --- | --- | --- | --- | --- | --- | --- | --- | --- | --- | --- |
|  |  | A |  | B |  | C |  | D |  | E |  |
| Age (years) | **<24** |  |  |  |  | **Ref.** |  |  |  |  |  |
|  | 24-27 | 0.72 | * | 0.72 | * | 0.70 | * | 0.71 | * | 0.72 | * |
|  | 28-34 | 0.61 | * | 0.61 | * | 0.60 | * | 0.61 | * | 0.62 | * |
|  | >34 | 0.37 | * | 0.37 | * | 0.36 | * | 0.37 | * | 0.37 | * |
| Gender identity | **Cisgender Women** |  |  |  |  | Ref. |  |  |  |  |  |
|  | Cisgender Men | 1.82 | * |  |  |  |  |  |  |  |  |
|  | Transgender Women | 1.72 |  |  |  |  |  |  |  |  |  |
|  | Transgender Men | 8.56 |  |  |  |  |  |  |  |  |  |
| Viral load (log copies/mL) ^b^ |  | 1.28 | * | 1.28 | * | 1.28 | * | 1.27 | * | 1.27 | * |
| CD4+ T cell count (cells/mm^3^) | **< 200** |  |  |  |  | **Ref.** |  |  |  |  |  |
|  | 200-500 | 1.77 | * | 1.73 | * | 1.70 | * | 1.70 | * | 1.72 | * |
|  | >500 | 1.86 | * | 1.80 | * | 1.76 | * | 1.75 | * | 1.79 | * |
| Sexual risk | **Cisgender Women** |  |  |  |  | **Ref.** |  |  |  |  |  |
|  | MSM |  |  | 2.47 | * | 2.15 | * | 2.29 | * | 2.61 | * |
|  | Transgender Women |  |  | 3.81 | * | 3.70 | * | 3.88 | * | 3.79 | * |
|  | Heterosexual Cisgender Men | |  | 1.63 |  | 1.40 |  | 1.57 |  | 1.71 |  |
|  | Missing |  |  | 2.58 | * | 1.20 |  | 1.16 |  | 2.57 | * |
| Venues for sex | **No** |  |  |  |  | **Ref.** |  |  |  |  |  |
|  | Yes |  |  |  |  | 1.17 |  | 1.21 |  | 1.21 |  |
|  | Missing |  |  |  |  | 1.04 |  | 1.05 |  | 1.13 |  |
| Education | **Elementary/none** | | | |  | **Ref.** |  |  |  |  |  |
|  | High school | |  |  |  | 1.21 |  | 1.18 |  |  |  |
|  | Career |  |  |  |  | 1.24 |  | 1.19 |  |  |  |
|  | Missing |  |  |  |  | 3.38 | * | 2.43 | * |  |  |
| Place where most time is spent | **Work** |  |  |  |  | **Ref.** |  |  |  |  |  |
|  | Home |  |  |  |  | 0.75 | * |  |  |  |  |
|  | School |  |  |  |  | 0.64 |  |  |  |  |  |
|  | Other |  |  |  |  | 1.37 |  |  |  |  |  |
|  | Missing |  |  |  |  | 0.56 |  |  |  |  |  |
|  | N | 3144 |  | 3144 |  | 3144 |  | 3144 |  | 3144 |  |
|  | LL_0 | -2059.00 |  | -2059.00 |  | -2059.00 |  | -2059.00 |  | -2059.00 |  |
|  | LL | -1968.68 |  | -1966.49 |  | -1958.58 |  | -1962.61 |  | -1965.61 |  |
|  | chi^2^ | 180.63 |  | 185.01 |  | 200.84 |  | 192.78 |  | 186.78 |  |
|  | r^2^_p | 0.04 |  | 0.04 |  | 0.05 |  | 0.05 |  | 0.05 |  |
|  | AIC | 3957.36 |  | **3954.98** |  | 3957.15 |  | 3957.22 |  | 3957.21 |  |
|  | BIC | 4017.90 |  | **4021.57** |  | 4078.22 |  | 4054.07 |  | 4035.91 |  |
|  | Hosmer-Lemeshow ^c^ | 0.13 |  | 0.62 |  | 0.24 |  | 0.66 |  | 0.55 |  |

MSM, men who have sex with men; Ref., reference category; LL, Log Likelihood; r2_p, pseudo R^2^; AIC, Akaike Information Criteria; BIC, Bayesian Information Criteria. ^a^ Odds ratio values of variables included in each model are shown. ^b^ Analyzed as continuous variable. ^c^ Hosmer-Lemeshow goodness of fit test p-value. * Statistically significant. Model B was selected as the most parsimonious.

**Supplementary Figure 1. Summary of characteristics of clusters with active growth.** Characteristics of the ten clusters for which at least one node was added in each time stage (trimester) are analyzed. Each panel shows a specific attribute of the participants in each cluster: gender, age, individuals that participate in links with genetic distance <0.5%, state of residence, CD4+ T cell count and grade (number of links per node).

**Supplementary Methods**

**2.2 HIV sequencing**

A fragment including the complete HIV *gag* and *pol* genes (5,462 bp; HXB2 positions: 769-6,231) was amplified and sequenced using standard next-generation sequencing (NGS) techniques. Briefly, viral RNA was extracted from 1 mL of plasma (QIAamp Viral RNA Kit; QIAGEN, Valencia, CA). For the first round of amplification, a one-step reverse transcriptase (RT)-PCR was performed in 12.5 μL final reaction volume, including 2.5 μL RNA, 0.2 μM of each primer: msf12b (forward, 5’-AAATCTCTAGCAGTGGCGCCCGAACAG-3’, [HXB2: 623-649]) and VIF-VPUoutR1 (reverse, 5’-GGTACCCCATAATAGACTGTRACCCACAA-3’, [HXB2: 6324-6352]), 0.25 μL of SuperScriptIII RT/Platinum Taq High Fidelity Enzyme (Invitrogen, Carlsbad, CA), 6.25 μL 2X Reaction Mix (containing 0.4 mM of each dNTP, 2.4 mM MgSO_4_) and 5% DMSO final concentration. Next, a second-round PCR was performed in 12.5 μL final reaction volume, with 1.25 μL of cDNA from the first round, 0.2 μM of each primer: F2nst (forward, 5’-GCGGAGGCTAGAAGGAGAGAGATGG-3’, [HXB2:769-793]) and VIF-VPUinR1 (reverse, 5’-CTCTCATTGCCACTGTCTTCTGCTC-3’, [HXB2: 6207-6231]), 0.375 μL Expand Long Template enzyme (Roche, Mannheim, Germany), 0.35 mM of each dNTP, 1.25 μL 10X ELT buffer (MgCl_2_ 17.5 mM) and 6% DMSO final concentration. The following conditions were used: 50°C for 30 min (first round only); 94°C for 2 min; 35 cycles of 94°C for 15 s, 55 °C for 30 s, 68°C for 6 min; and a final extension of 68°C for 5 min. Genetic libraries were produced and barcoded by standard methods (Nextera XT, Illumina, San Diego, CA). Libraries were pooled and sequenced with paired-end 500-cycle cartridges on a MiSeq instrument (Illumina). Reads were filtered and assembled using the publicly available program HyDRA Web (Public Health Agency of Canada) [31, 32]. Twenty percent consensus sequences were generated and used in HIV drug resistance and clustering analyses. This threshold has been previously defined to provide an excellent agreement between NGS and standard Sanger sequencing [33]. The sequence database was curated, excluding duplicates, as well as sequences not compliant with quality control [30]. Briefly, sequences were excluded due to inadequate length, presence of stop codons, bad insertions/deletions, excess APOBEC or unusual mutations.

**2.3 Clustering analyses**

Clusters were defined by a genetic distance matrix method, using Seguro HIV-TRAnsmission Cluster Engine (Seguro HIV-TRACE), a locally adapted and secured version of the widely-used HIV-TRACE tool [34]. Seguro HIV-TRACE, like the Secure HIV-TRACE implemented for U.S. public health departments, permits the analysis and storage of HIV transmission clusters accessible only to registered users. The HIV-TRACE display was translated into Spanish and adapted to include the variables of interest for the study and fulfill the local data security requirements. Briefly, Seguro HIV-TRACE establishes and administers privileged user accounts in accordance with a role-based access scheme that organizes allowed information system access and privileges into roles. Roles included are: User, Executive (Read-only access), Service Administrator (IT Support with access to multiple site accounts), and Administrator. Additionally, users may be assigned “Site Administrator”, which allows site provisioning, such as creating or removing users for their respective site. Privileged role assignments are monitored through the admin portal. Activity, such as time of last log-in and site assignment, is reported. System logs also record time since last password change, and http requests to the site. There is no publicly available content on Seguro HIV-TRACE, and interaction with the system requires a user account. Clusters were defined as sequences with pairwise Tamura-Nei 93 genetic distance <0.015. This threshold has been previously demonstrated to be in line with the expected divergence between sequences within an individual [35] and in accordance with the genetic distance between named HIV risk partners [36, 37]. Georeferencing of participants according to municipality and zip code of residence was performed according to National Institute of Statistics and Geography (INEGI) coding, using the program QGIS v3.16.
